# A Comparison of p53 Isoform Profiles and Apoptosis Induced by Camptothecin or a Herbal Khat Extract (*Catha Edulis (Vahl) Forssk. ex Endl.*) in Leukemic Cell Lines: Exploring Cellular Responses in Therapy Development

**DOI:** 10.3390/cancers12123596

**Published:** 2020-12-01

**Authors:** Therese Bredholt Onyango, Sigrun M. Hjelle, Ingvild Haaland, Olav K. Vintermyr, Anne Christine Johannessen, Bjørn Tore Gjertsen

**Affiliations:** 1 Centre for Cancer Biomarkers CCBIO, Department of Clinical Science, University of Bergen, N-5021 Bergen, Norway; Therese.Bredholt@uib.no (T.B.O.); sigrun.margrethe.hjelle@helse-bergen.no (S.M.H.); Ingvild.Haaland@uib.no (I.H.); 2 Influenza Centre, Department of Clinical Science, University of Bergen, N-5021 Bergen, Norway; 3 Department of Pathology, Haukeland University Hospital, N-5021 Bergen, Norway; Olav.Vintermyr@uib.no (O.K.V.); Anne.Johannessen@uib.no (A.C.J.); 4 The Gade Laboratory for Pathology, Department of Clinical Medicine, Haukeland University Hospital, N-5021 Bergen, Norway; 5 Department of Medicine, Hematology Section, Haukeland University Hospital, N-5021 Bergen, Norway

**Keywords:** p53 isoforms, PTMs, CPT, khat

## Abstract

**Simple Summary:**

This study aimed at exploring the modulations of p53 in cell toxicity induced by an extract of the herb khat, a natural stimulant used by millions of people. We previously reported that khat-extract induced cell death by affecting mitochondrial function and the receptor- and mitochondria-mediated cell death pathways, in leukemic cell lines and cells of the oral cavity, in vitro. We included the cancer therapeutic camptothecin, which induces apoptosis in various cancer cell lines. By studying modulations of p53 full-length protein and p53 β/γ isoforms following exposure to khat-extract and camptothecin, we wished to elucidate differences and similarities resulting from the treatments using MOLM-13 and MV-4-11 leukemic cell lines. Our results demonstrate that molecular effects of the cytotoxic treatments resulted in different p53 isoforms patterns and post-translational modifications. We suggest that analysis of p53 modulations could be useful in the search for new chemical probes and experimental cancer therapeutics.

**Abstract:**

Khat (*Catha edulis (Vahl) Forssk. ex Endl*.) is habitually used as a natural stimulant by millions of people, but is associated with adverse effects on gastrointestinal, cardiovascular and central neural systems. At the cellular level khat toxicity involves p53 induction and cell cycle arrest, decreased mitochondrial function and activation of receptor- and mitochondria-mediated cell death pathways. In this study we have examined an extract of khat for induction of p53 post-translational modifications (PTMs) and the functional role of p53 in khat-mediated cell death. Khat was shown to induce phosphorylation and acetylation of p53 in both the khat-sensitive MOLM-13 and the khat-resistant MV-4-11 cell line, but accumulation of the full-length p53 isoform was only observed in the khat sensitive cell line. Small molecule inhibitors of p38 MAP kinase sensitized MV-4-11 cells for khat-treatment without concomitant stabilization of p53. Experiments using a p53 knock-down cell line and murine p53 knock-out bone marrow cells indicated that p53 was redundant in khat-mediated cell death in vitro. We suggest that analysis of isoform patterns and p53 PTMs are useful for elucidation of biological effects of complex plant extracts, and that p53 protein analysis is particularly useful in the search for new chemical probes and experimental cancer therapeutics.

## 1. Introduction

p53 acts as a stress sensor and guardian of the genome in healthy cells, and its prominent role in carcinogenesis is illustrated by its frequent mutational rate in solid cancers [1,2]. Mutated p53 is associated with chemoresistant disease and the wild type p53 protein regulates cellular processes like proliferation, differentiation and induction of programmed cell death.

In healthy cells p53 levels are kept low in non-stressed cells by the human double minute protein, Mdm2, which functions as a p53-specific E3 ubiquitin ligase [3,4]. Mdm2-mediated mono-ubiquitination precedes poly-ubiquitination, which targets p53 for proteasomal degradation [5]. The p53 family members p63 and p73 both have overlapping and distinct functions, and are also regulated by the ubiquitin proteasomal pathway [6].

Various stress stimuli induce post-translational modifications (PTMs) of p53 including phosphorylation and acetylation, which mediate stabilization and accumulation of the p53 protein [7]. In addition to being regulated by PTMs, the function of p53 is directed by alternative mRNA splicing and expression of various isoforms [8,9]. When meditating cell death, p53 may act as a sequence specific transcription factor inducing pro-apoptotic genes, but also triggers cell death through transcription-independent activities, e.g., through interaction with Bcl-2 family members in the outer mitochondrial membrane [10]. 

The herb khat (*Catha edulis (Vahl) Forssk. ex Endl*.), is chewed for its stimulating potential by millions of people and habitual use is associated with adverse effects on various organ systems, including the gastrointestinal, cardiovascular and the central nervous system [11,12]. We previously reported that an extract of khat induced programmed cell death in leukemic cell lines with and without p53, affecting mitochondrial function and proteins of the receptor- and mitochondria-mediated cell death pathways [13,14]. Further, we demonstrated induction of p53 and cell cycle arrest in normal oral keratinocytes and fibroblasts [15]. In this study we examined the molecular modulations and functional role of p53 in khat-treated acute myeloid leukemia (AML) cell lines sensitive (MOLM-13) or resistant (MV4-11) to khat-induced apoptosis. Khat was shown to induce phosphorylation and acetylation of p53 in both cell lines, but accumulation of the full-length (FL) isoform, p53 FL, was only seen in MOLM-13. Inhibition of p38 MAP kinase sensitized MV-4-11 cells towards khat, apparently without concomitant stabilization of p53. Experiments using p53 knock-down and knock-out cells confirmed that p53 was mostly redundant in khat-mediated cell death in vitro. Analysis of isoform patterns and p53 PTMs appear attractive in the elucidation of mechanisms of action of complex plant extracts.

## 2. Results

### 2.1. Khat Reduced MOLM-13 Proliferation/Viability in a Concentration Dependent Manner

**Figure 1:**
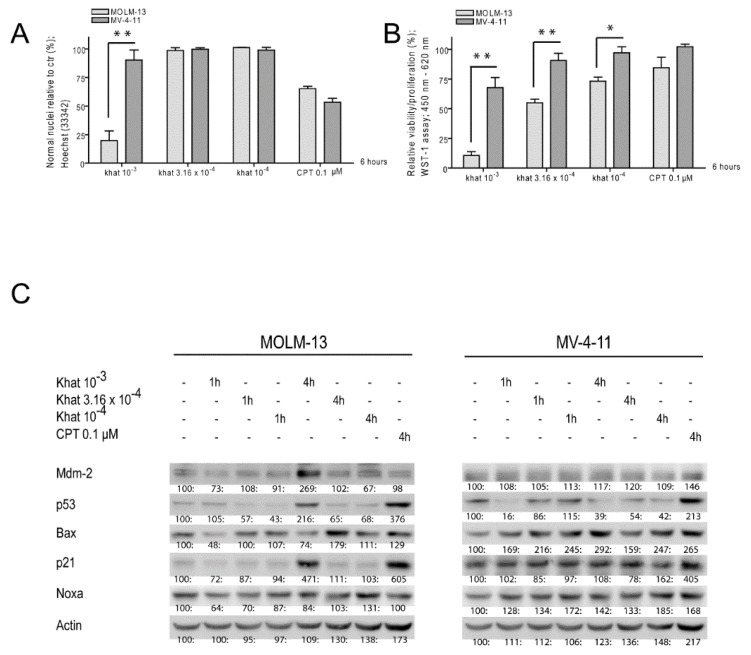
Induction of p53 and transcriptional targets in khat-sensitive MOLM-13 cells. MOLM-13 and MV-4-11 were exposed to khat-extract dilutions of 10^–3^, 3.16 × 10^–4^ and 10^–4^, and to 0.1 μM camptothecin (CPT). (**A**) Effects on nuclear morphology were evaluated after 6 h following Hoechst staining using epifluorescent microscopy. The results are presented as percentages of cells with normal nuclear morphology as compared to controls. The experiments were run in triplicates and repeated three times. The asterisks represent levels of statistical significance, with ** = *p* ≤ 0.01. (**B**) Effects on cell viability/proliferation were assessed after 6 h using the WST-1 assay. The results were collected by measuring absorbance (450–620 nm) and are presented as: [(absorbance treated cells)/(absorbance control cells)] × 100. The experiments were run in triplicates and repeated three times. The asterisks represent levels of statistical significance, with * = *p* ≤ 0.05 and ** = *p* ≤ 0.01. (**C**) Alterations in levels of p53 and p53 target proteins were evaluated after 1 and 4 h by one-dimensional polyacrylamide gel electrophoresis (1D-PAGE) and Western blot (original blots can be found at Figure S1). β-actin was used as loading control. The intensity of each band was evaluated using the ImageJ software, and are presented as a ratio: [(intensity of treated sample)/(intensity of control sample)] × 100. The experiments were repeated three times.

A 10^–3^ dilution of an organic extract of khat was shown to induce cell death in various AML cell lines, based on alterations in nuclear morphology [13,14]. The time of incubation selected for analysis of early molecular events was based on our previous in vitro and in vivo studies of both khat-extract and chemotherapy [13,16]. The cancer therapeutic camptothecin (CPT) was included to evaluate the toxic potential of khat, since CPT represents a defined botanical alkaloid that causes topoisomerase I-linked DNA breaks and apoptosis in various cancer cell lines [17,18]. CPT (0.1 μM) induced comparable levels of cell death in the two cell lines (Figure 1A). We exposed the khat-sensitive MOLM-13 and the khat-resistant MV-4-11 leukemic cell lines to different khat-extract dilutions from 1 to 6 h. At 6 h only the highest khat concentration induced cell death in MOLM-13 cells, whereas MV-4-11 appeared resistant to all khat concentrations (Figure 1A). 

Cytotoxic effects of khat and CPT were further evaluated with the WST-1 viability/proliferation assay. The assay relies on cleavage of a tetrazolium salt to soluble formazan by complex II in the mitochondrial respiratory chain [19]. The highest khat concentration strongly impaired MOLM-13 viability/proliferation, whereas MV-4-11 was only partly affected. The lower khat concentrations caused reduced viability/proliferation of MOLM-13 in a concentration dependent manner (Figure 1B).

### 2.2. Khat Induced p53 Protein in MOLM-13 but Temporary Attenuated p53 in MV-4-11 

MOLM-13 and MV-4-11 were exposed to khat-extract dilutions of 10^–3^, 3.16 × 10^–4^ and 10^–4^, in addition to 0.1 μM CPT. The cell samples were analyzed for expression levels of p53 and selected p53 target proteins by one-dimensional polyacrylamide gel electrophoresis (1D-PAGE) and Western blotting (Figure 1C). The khat concentration found to mediate cell death and reduced viability/proliferation (10^–3^) significantly induced p53 in MOLM-13 after 4 h, whereas a temporary p53 down-regulation was observed in MV-4-11. Mdm2 and p21 were induced in MOLM-13, whereas their levels remained unaltered in MV-4-11. CPT induced p53 and p21 in both MOLM-13 and MV-4-11. 

### 2.3. Khat Altered p53 Isoform Distribution and Induced Post-Translational Modifications

**Figure 2:**
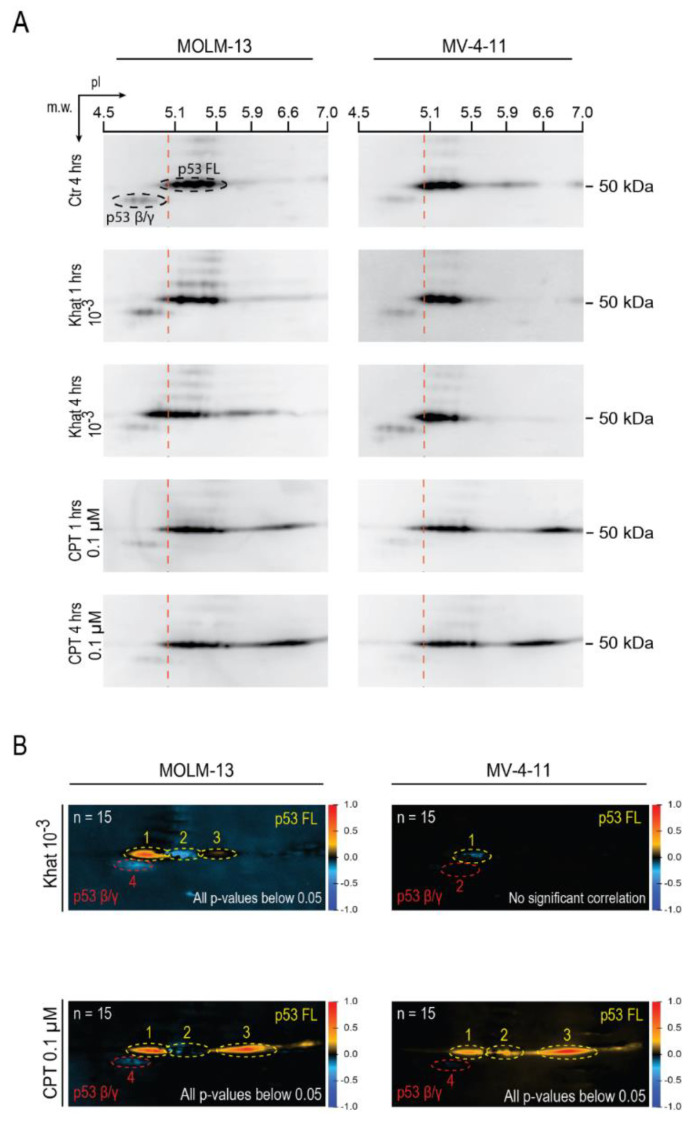
Khat induced post-translational modifications (PTMs) and altered p53 isoform patterns. (**A**) MOLM-13 and MV-4-11 were exposed to a khat-extract dilution of 10^–3^ and 0.1 μM CPT for 1 and 4 h, and analyzed for alterations in p53 isoform patterns using two-dimensional polyacrylamide gel electrophoresis (2D-PAGE) and Western blotting (original blots can be found at Figure S2). (**B**) Three separate experiments, resulting in 15 2D-PAGE Western blots (*n* = 15), were included in a correlation analysis [22]. The resulting correlation image depicts the correlation to the external parameter; hours of treatment with khat or CPT, with colors varying from red (positive correlation) through black (no correlation) to blue (negative correlation). Correlation values were collected from defined regions of interest and used to calculate the *t*-value [22], and the probability of significance (*p*-value) was found by calculating the *t*-distribution in a two-tailed Student’s *t*-test with n–1 degrees of freedom.

Only the 10^–3^ khat dilution induced p53 in MOLM-13 at 1 and 4 h, whereas p53 appeared temporarily down-regulated in MV-4-11. To further study these changes, p53 was analyzed by two-dimensional polyacrylamide gel electrophoresis (2D-PAGE) and Western blotting techniques [20,21]. The 2D blots of MOLM-13 demonstrated that the p53 FL isoform became increasingly more acidic by khat treatment over time (Figure 2A). 

**Figure 3:**
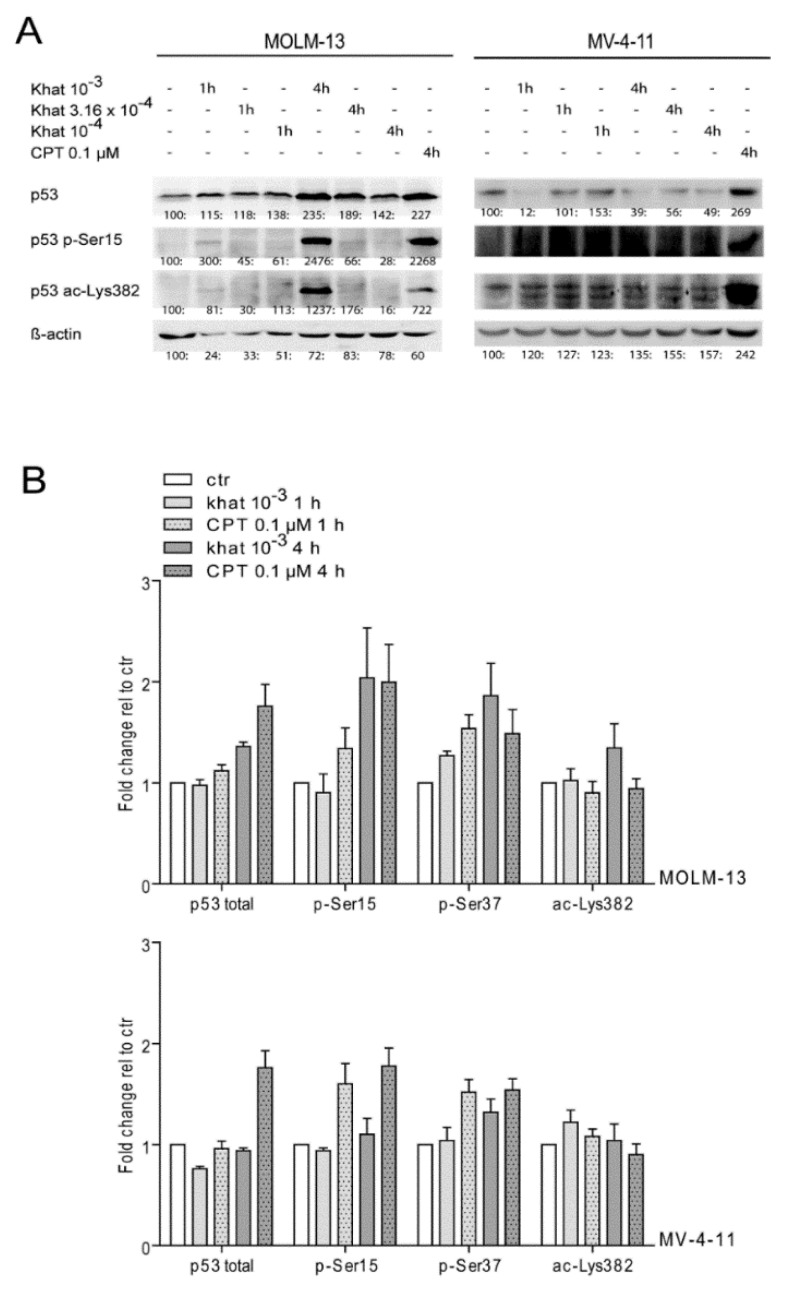
Khat induced p53 phosphorylation and acetylation in MOLM-13 and MV-4-11. (**A**) MOLM-13 and MV-4-11 were exposed to khat-extract dilutions of 10^–3^, 3.16 × 10^–4^ and 10^–4^, and to 0.1 μM CPT. Alterations in levels of total p53 and p53 phosphorylated at serine 15 and acetylated at lysine 382 were evaluated after 1 and 4 h by 1D-PAGE and Western blotting techniques (original blots can be found at Figure S3). β-actin was used as loading control. The intensity of each band was evaluated using the ImageJ software, and are presented as a ratio: [(intensity of treated sample)/(intensity of control sample)] × 100. (**B**) MOLM-13 and MV-4-11 were exposed a khat-extract dilution of 10^–3^ and 0.1 μM CPT for 1 h before flow cytometric analysis of total p53, p53 phosphorylated at serine 15, serine 37 and acetylated at lysine 382. Results are given as % of control in median fluorescence intensity.

The 2D blots were analyzed using used a method that was developed to enable correlation of individual images to biological parameters [22]. The acidic shift in the 2D blots was verified in the resulting correlation image, where the red and blue colors indicate positive and negative correlations to khat treatment, respectively (Figure 2B). The shift suggested increased levels of phosphorylation and/or acetylation, which will add negative charges to the p53 protein. The levels of p53β and p53γ isoforms at approximately 48 kDa appeared reduced in the khat-treated MOLM-13 cells. The khat-mediated changes were less pronounced in MV-4-11 (Figure 2B). A general acidification of p53 appeared in form of relocation of the p53 distribution toward more acidic forms. This lack of increase in total p53, but increase in phosphorylated and acetylated p53, was confirmed by intracellular flow cytometry of p53 and its PTMs (Figure 3B). Our in-house correlation technique confirmed the initial down regulation of p53 (Figure 2B).

### 2.4. Khat Induced Phosphorylation and Acetylation of p53 in MOLM-13 and MV-4-11

Specific p53 PTMs were analyzed in MOLM-13 and MV-4-11 exposed to 10^–3^, 3.16 × 10^–4^ and 10^–4^ dilutions of khat and 0.1 μM CPT by 1D-PAGE and Western blotting (Figure 3A). p53 was shown to be phosphorylated at serine 15 and acetylated at lysine 382 in MOLM-13 treated with the 10^–3^ dilution of khat. The Western blot images of khat-treated MV-4-11 cells did not indicate PTMs of p53. 

**Figure 4:**
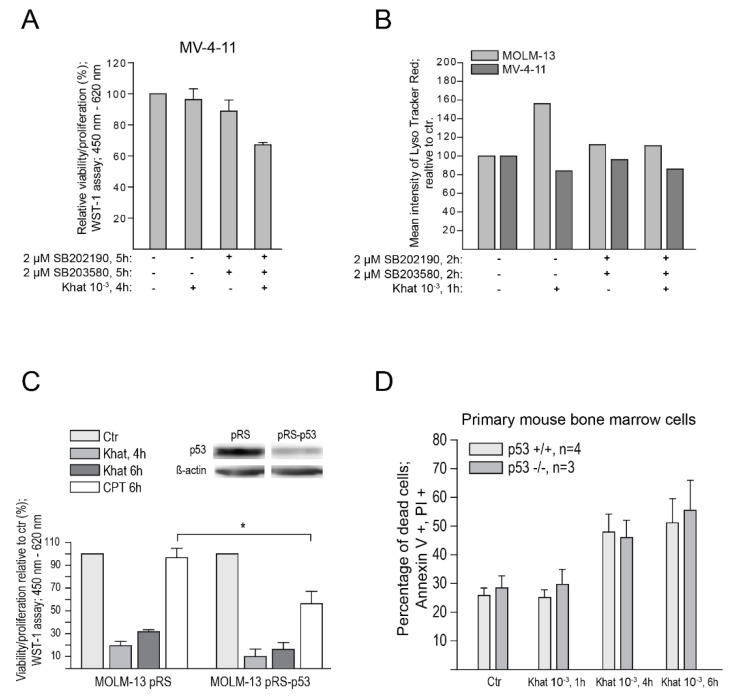
p38 inhibitors sensitized resistant MV-4-11 cells to khat and p53 are not necessary for khat-cytotoxicity. (**A**) MV-4-11 cells were exposed to a khat-extract dilution of 10^–3^ for 4 h and/or pre-treated with p38 inhibitors for 1 h (SB203580, 2 µM, and SB202190,2 µM). Effects on cell viability/proliferation were assessed after 4 h using the WST-1 assay. The results were collected by measuring absorbance (450–620 nm) and are presented as: [(absorbance treated cells)/(absorbance control cells)] × 100. (**B**) MOLM-13 and MV-4-11 were exposed to a khat-extract dilution of 10^–3^ for 1 h and/or pre-treated with p38 inhibitors for 1 h (SB203580, 2 µM, and SB202190, 2 µM). Lyso Tracker^®^ Red was added to the cell culture medium the last 30 min of khat-exposure, the cells were washed and fixed before analysis by flow cytometry. Results are shown as mean fluorescence intensities relative to the control samples. (**C**) MOLM-13 wt and MOLM-13 shp53 cells were exposed to a khat-extract dilution (10^–3^) and 0.1 µM CPT for 4 and 6 h. Effects on cell viability/proliferation were assessed after 4 and 6 h using the WST-1 assay. The results were collected by measuring absorbance (450–620 nm) and are presented as: [(absorbance treated cells)/(absorbance control cells)] × 100. Knock down of p53 in the shRNA transduced MOLM-13 cells was verified by Western blotting. β-actin was used as loading control. The asterisk represent levels of statistical significance, with * = *p* ≤ 0.05. (**D**) Bone marrow from p53 −/− mice and their wild-type littermates were harvested from femurs, subjected to red cell lysis and washed before exposure to a khat-extract dilution (10^–3^) for 1, 4 and 6 h. Cell death was assessed using Annexin-V FITC and propidium iodide (PI) in a flow cytometry analysis.

The introduction of PTMs was in addition analyzed using p53-modification specific antibodies and intracellular flow cytometry of MOLM-13 and MV-4-11 cells that had been exposed to the 10^–3^ dilution of khat for 1 h. The analyses demonstrated increased levels of p53 phosphorylated at serine 15 and 37, and acetylated at lysine 382, in both the MOLM-13 and MV-4-11 cell line (Figure 3B). We examined cell cycle distribution in khat-treated MV-4-11 (10^–3^ dilution) by flow cytometry at 24 and 48 h, and observed normal distribution between G1/M/S phase (see [15] for experimental procedure). The unaffected cell cycle in MV-4-11 was consistent with the lack of p21 induction (Figure 1C; 4 h) and normal cell proliferation observed in MV-4-11 cells cultured in presence of khat-extract (48–72 h). We conclude that khat-induced p53 modulation observed in MV-4-11 did not impact proliferation or cell death in this cell line. 

### 2.5. p38 Inhibitors Sensitized Resistant MV-4-11 Cells to Khat

Lukandu et al. [23] observed that khat-induced cell death involved p38-activation and that inhibition of p38 protected against khat-toxicity. Interestingly the khat-resistant cell line MV-4-11 contains a K-Ras mutation that mediates constitutive activation of p38 [24]. Since p38 acts upstream of p53, we thus tested if p38 inhibition could sensitize MV-4-11 cells to khat. MV-4-11 was pre-treated with the p38 inhibitors SB202190 and SB203580 and exposed to the khat for 4 h. Cytotoxic effects were assessed with the WST-1 assay (Figure 4A). A significant reduction in cell viability was seen upon pre-treatment with p38 inhibitors, supporting a role for p38 in khat sensitivity. Analysis of p53 by flow cytometry (1 h of incubation) and 2D-PAGE (up to 4 h of incubation with khat, 1 h pre-incubation with p38 inhibitors) indicated no increased stabilization of p53 in MV-4-11 even if the cell line was sensitized for khat-induced apoptosis. 

We previously reported that khat mediated morphological characteristics of autophagy in MOLM-13 cells [13]. In order to elucidate the involvement of the autophagosomal–lysosomal pathway in khat-induced toxicity in MOLM-13 and MV-4-11, we performed flow cytometric quantification of lysosomes and/or their acidification (Lyso Tracker^®^ Red DND-99, Invitrogen, Inc., Waltham, MA, USA). MOLM-13 and MV-4-11 cells were treated with the 10^–3^ dilution of khat, with or without pre-treatment with p38-inhibitors (Figure 4B). In agreement with our previous observations of khat-triggered autophagy, we observed an increase in lysosome acidification in khat-treated MOLM-13 cells. Further, the results demonstrated that pre-treatment of MOLM-13 with p38 inhibitors abolished this activation. In contrast, none of the treatments appeared to activate the autophagosomal–lysosomal compartment in MV-4-11. This could indicate that khat-induced autophagy depends on functional p53 of MOLM-13, consistent with an important role for p53 in the regulation of autophagy [25]. An alternative explanation may be that MV-4-11 has a defective mechanism for autophagy, independent of p53 function.

### 2.6. Functional p53 Protein Is Not Essential for Khat-Mediated Cell Death 

In spite of p53 protein activation in khat induced cell death (Figure 1) we have previously observed p53 independence of khat toxicity when examined in various cell lines [13,14]. Inhibiting p38 signaling led to increased sensitivity of MV-4-11 cells towards khat, with no changes in p53 isoform distribution (Figure 4A). In order to establish the functional role of p53 activation in khat-mediated cell death, we tested cells with deleted or reduced levels of p53. Short hairpin (sh) RNAs against p53 was transduced into MOLM-13 cells generating the MOLM-13 shp53 cell line with reduced levels of wild type p53 (Figure 4C). When exposing the two cell lines to khat, no significant difference could be seen in cell death levels between MOLM-13 wt and MOLM-13 shp53 (Figure 4C; *p* = 0.08 control versus 4 h of khat). These results indicated that khat-mediated cell death was regulated upstream or independently of p53. Since khat has been reported to be toxic to bone marrow [26], we examined bone marrow cells from p53 −/− mice and their wild-type littermates exposed to 10^–3^ dilution of khat in vitro and evaluated cell death-induction (Figure 4D). Cell-death was induced in both p53 −/− cells and in the wild type cells to the same extent. Both the MOLM-13 cell line with down regulated p53 and the bone marrow cells with knocked out p53 demonstrated a non-significant tendency of decreased khat sensitivity after a 4 h incubation. This observation suggested a minor role for p53 in khat-mediated cell death in vitro.

## 3. Discussion 

In this study we have explored the effect of khat treatment on induction of p53 PTMs, on p53 isoform distribution and evaluated the contribution of p53 in khat-mediated cell death. As previously reported, khat was shown to mediate cell death in MOLM-13, whereas MV-4-11 was relatively resistant [13]. The p53 FL isoform was acidified and shown to accumulate in MOLM-13 cells, whereas the levels of truncated β/γ p53 isoforms were reduced (Figure 2A). This observed p53 isoform modulation was previously reported in AML patients receiving induction chemotherapy [21]. p53 FL is believed to represent an activated version, whereas the truncated isoforms have been suggested to function as negative regulators of p53 activity [9]. Hence, alterations in p53 isoform distribution indicated increased activity of p53, suggesting its involvement in khat-mediated cell death. 

The p53 isoform distribution suggested reduced levels of both p53 FL and the truncated isoforms in khat-treated MV-4-11. However, the 2D analyses indicated introduction of PTMs, mediating a slight acidification of p53 FL in MV-4-11. The use of flow cytometric analyses confirmed p53 phosphorylation and acetylation in both MOLM-13 and MV-4-11 cells following treatment with khat. Phosphorylation and acetylation are known to mediate stabilization and accumulation of p53, but stabilization of p53 was not observed in MV-4-11 (Figure 3B). Induction of p53 phosphorylation in absence of elevated p53 levels has previously been reported in studies on plant extracts [27].

Several cell line characteristics of MV-4-11 could account for its resistance to khat-mediated cell death, including a mutation in the *K-RAS* gene [28]. The mutated K-Ras protein has been shown to inhibit the function of p53 and activate the PI3K/AKT pathway [29,30]. The K-Ras protein mutation could further mediate increased levels of ROS and constitutive activation of p38 [24]. Khat has been shown to mediate p53 activation in normal oral keratinocytes and fibroblasts [15], and activation of the protein kinase p38 was reported [23]. Further, p38 activation and khat cytotoxicity was shown to be inhibited by pre-incubation with specific p38 inhibitors. 

We hypothesized that p38 inhibitors could sensitize MV-4-11 to khat, by attenuating the constitutive p38 activation in this cell line. In agreement with this, MV-4-11 was observed to be sensitized to khat (Figure 4A) but without stabilization of the p53 protein. However, the p38 inhibitor SB202190 has been shown to stimulate growth of the MV-4-11 cell line [29], and increased proliferation has been shown to sensitize leukemia cells to drug-induced cell death [30]. It is therefore possible that the p38 inhibitors enhanced the toxic effect of khat by stimulating cell proliferation.

We previously reported that khat induced morphological characteristics of autophagy in MOLM-13 cells [13]. Here, we used Lyso Tracker^®^ Red to measure the level of acidic compartments like autolysosomes, which are fusions of lysosomes and autophagosomes. The results further supported involvement of the autophagosomal–lysosomal compartment in khat-mediated cell death in MOLM-13 (Figure 4B). Interestingly, pre-treatment with the p38 inhibitors abolished activation of this pathway (Figure 4B). p38 is activated upstream of p53, and the p38 inhibitors could therefore inhibit activation of p53, which has the potential to trigger autophagy. However, p53 is reported to play a dual role in autophagy, with nuclear p53 inducing autophagy genes, whereas cytoplasmic p53 represses its activation [31]. In contrast to the MOLM-13 cells, the autophagosomal–lysosomal pathway did not appear to be activated in MV-4-11. 

There are several indications of p53 acting primarily as a stress sensor as opposed to being an inducer of khat-mediated cell death in MOLM-13. First, p53 is significantly induced at a relatively late time point, p53 does not induce typical pro-apoptotic proteins like Bax and Noxa, and most important: the p53 knock-out/knock-down cells demonstrated that p53 was not necessary for cell death-induction (Figure 4C,D). Further, the AML cell lines NB4 and HL60 which have mutated/deleted TP53, were previously shown to be sensitive to khat-mediated cell death [13]. 

## 4. Materials and Methods

### 4.1. Khat (Catha edulis (Vahl) Forssk. ex Endl.) Extract

Khat samples from the Meru district in Kenya were extracted using methanol and a dimethylsulfoxide (DMSO; Sigma, St. Louis, MO, USA) stock solution was prepared as previously described [13,14]. The stock solution was diluted 1:10 in RPMI 1640 medium (Sigma) with 10% foetal bovine serum (FBS) and antibiotics (2mM L-glutamine, 100 IU/mL penicillin and 100 μg/mL streptomycin; Gibco, Waltham, MA, USA), and precipitates were removed by centrifugation (10,000× *g*, 15 min, 4 °C). The khat supernatant was added to experimental cell cultures giving final dilutions of 10^–3^, 3.16 × 10^–4^ and 10^–4^. The DMSO concentration in experimental cell cultures was 0.1% and control cells were added an equivalent amount of DMSO. 

The concentrations of S-(-)-cathinone, (1S,2S)-(-)-cathine and (1R,2S)-(-)-norephedrine in the khat-extract were determined using high pressure liquid chromatography and mass spectrometry (HPLC-MS-MS), as previously described [13]. The alkaloid concentrations were: cathinone: 2.5; cathine: 3.0 and norephedrine: 0.3; all values presented as mg/mL. 

### 4.2. Chemicals

Camptothecin (CPT) was purchased from Sigma-Aldrich, the p38 inhibitors SB202190 from SA Biosciences, Frederick, MD, USA and SB203580 from BioSource, Nivelles, Belgium.

### 4.3. Cell Lines and Cultivation

MV-4-11 was purchased from ATCC (American Type Culture Collection, Manassas, VA, USA) and cultured in IMDM medium (BioWhittaker, Cambrex Bio Science, Verviers, Belgium) with 10% FBS and antibiotics. MOLM–13 was a generous gift from Dr. Kunzo Orita, Okayama, Japan, cultured in RPMI 1640 medium (Sigma) with 10% FBS and antibiotics. MV-4-11 carries a TP53 point mutation in one allele (CAT→CGT at codon 344, exon 9) [32], while MOLM-13 expresses wild type p53. p53 knocked down MOLM-13 cells were generated by retroviral transduction. Phoenix virus producer cell line was co-transfected with pRETRO-super or pRETRO-SUPER-p53 [33] and pCI-VSVG, with the calcium phosphate method. MOLM-13 cells were infected with viruses and increases in concentration were facilitated by centrifugation (50,000× *g*, 3 h). A total of 48 h after virus infection, the transfected cells were selected with puromycin. The concentration of puromycin was gradually increased up to 400 µg/mL within two weeks. Knock down was verified with Western Blot.

The cell lines were routinely screened for mycoplasma and maintained in a humidified atmosphere at 37 °C with 5% CO_2_. When used in experiments, the cell lines were seeded at a concentration of 200,000 cells/mL.

### 4.4. Cell Death Determination Based on Nuclear Morphology

Cell death was determined as described previously by microscopy of cell aliquots fixed in nutrient media containing 4% formaldehyde with 10 μg/mL of the DNA-specific fluorochrome, bisbenzimide (Hoechst 33342; Calbiochem, San Diego, CA, USA) [34].

### 4.5. Cell Viability/Proliferation Assay

Viability/proliferation was determined using the Cell Proliferation Reagent WST-1 (Roche Applied Science, Penzberg, Germany) according to the manufacturer’s protocol. Aliquots (100 μL) of cells (200,000/mL) were added 10 μL WST-1 two hours after initiation of the experiments. The cells were incubated for 2–4 additional hours and the results collected using a Tecan Infinite 200 microplate reader and Magellan software (version 6) (Tecan Trading AG, Männendorf, Switzerland). The results are presented as percentage viability of treated cells relative to controls, using the following formula: [(abs. treated cells)/(abs. control cells)] × 100.

### 4.6. Animal Experiments

All experiments were approved by the Norwegian Animal Research Authority and conducted according to the European Convention for the Protection of Vertebrates used for Scientific Purposes, in compliance with the EU Directive 2010/63/EU on the Protection of Animals used for Scientific Purposes.

C57Bl/6 and p53 −/− mice [35] were a generous gift from Prof. Guillermina Lozano (Department of Genetics, The University of Texas MD Anderson Cancer Center, Houston, TX, USA) and Prof. Jean Christophe Marine (Laboratory for Molecular Cancer Biology, VIB-UGent, Ghent B-9052, Belgium). They were bred and maintained under defined flora conditions in a high-efficiency particulate arrester-filtered atmosphere. Bone marrows were harvested from femur, washed in PBS before red blood cells were lysed using BD Pharm Lyse™ lysing solution according to the manufacturer’s instructions. The cells were seeded in RPMI 1640 with 10% FBS and antibiotics at a concentration of 1 × 10^6^/mL. 

### 4.7. Measurement of Cell Death in Primary Mouse Bone Marrow Cells

Cell death was determined with dual color flow cytometry using Annexin-V FITC and PI (APOTEST-FITC, Nexin Research, Kattendijke, The Netherlands), as described in [36].

### 4.8. Flow Cytometry

For p53 phospho/acetyl-flow, cells were washed in PBS, fixed in BD Cytofix Fixation buffer (BD Biosciences, San Jose, CA, USA), and permeabilized in 90% methanol in PBS. Cells were washed in 0.5% BSA in PBS and stained with FITC Mouse Anti-Human p53 (BD), Phospho-p53 (Ser15) (16G8) Mouse mAb Alexa Fluor 488 conjugate (Cell Signaling Technologies, Beverly, MA, USA), Alexa Fluor 647 Mouse anti-p53 (pS37) (BD), or Acetyl-p53 (Lys382) Antibody (Cell Signaling Technology, Danvers, MA, USA) followed by secondary Alexa Fluor 647 F(ab’)2 fragment of goat anti-rabbit IgG (H+L) (Invitrogen Corp., Carlsbad, CA, USA).

Lyso Tracker^®^ Red DND-99 (Invitrogen) was used to look for features of autophagic cell death. Lyso Tracker was diluted to working solution as indicated by the supplier and incubated with the cells for 30 min. Cells were washed in PBS and fixed in 1% PFA. Red fluorescence was measured by flow cytometry (FACS Calibur, Becton Dickinson, San Jose, CA, USA). Data were analyzed using FlowJo software (Tree Star, Inc., Ashland, OR, USA). Results are shown as mean fluorescence relative to the control samples.

### 4.9. One-Dimensional Polyacrylamide Gel Electrophoresis (1D-PAGE) and Western Blotting

Cells (4 × 10^6^ pr experimental condition) were washed with ice cold NaCl (9 mg/mL) and proteins resolved by gel electrophoresis (12.5% gels) under denaturating conditions and analyzed by Western blotting, as previously described [37]. The membranes were probed with antibodies against Bax (2D2), Mcl-1 (22), Noxa (FL-54), MDM2 (SMP-14), p53 (Bp53-12), purchased from Santa Cruz Biotechnology, Dallas, TX, USA, and against Puma (Cell Signaling Technology), p21 (BD Pharmingen) and acetyl-p53 (Lys382), phospho-p53 (Ser15) (16G8), the p53 antibodies were from Cell Signaling Technology. Anti-β-actin (mAbcam no. 8226; Abcam Inc., Cambridge, MA, USA) was used as loading control. Anti-mouse and anti-rabbit IgG secondary antibodies conjugated to Horseradish Peroxidase (Jackson ImmunoResearch, West Grove, PA, USA) were detected by enhanced chemiluminescence (Supersignal West Pico, Pierce Biotechnology, Rockford, IL, USA) and visualized using a KODAK Image Station 2000R (Eastman Kodak Company, Lake Avenue, Rochester, NY, USA). The intensity of each band was evaluated using the ImageJ software, and are presented as a ratio: [(intensity of treated sample)/(intensity of control sample)] × 100.

### 4.10. Two-Dimensional Polyacrylamide Gel Electrophoresis (2D-PAGE)

Cells (4 × 10^6^) were washed with cold saline (9 mg/mL), pelleted by centrifugation (1800 rpm, 7 min, 4 °C) and lysed in 1 mL 7% trichloroacetic acid (TCA). The protein precipitate was pelleted by centrifugation (13,000 rpm, 30 min, 4 °C), washed in 5%TCA and desalted with water saturated ether. The protein pellet was dissolved in 2D-sample buffer (7M urea, 2M thiourea, 100 mDTT, 1.5% Ampholyte 5-6, 0.5% CHAPS) and total protein concentration determined using the Bradford assay. The proteins were subjected to two-dimensional polyacrylamide gel electrophoresis (2D-PAGE) and Western blotting, as previously described [37,38]. p53 was detected using primary BP53-12 antibodies (sc-263; Santa Cruz Biotechnology) and secondary Horseradish Peroxidase-conjugated anti-mouse antibodies (Jackson ImmunoResearch) and the protein pattern visualized as described in the previous section. 

The p53 2DI images were analyzed using the correlation software Gel2DE v. 1.8 [22,39]. The 2DI images were normalized and aligned after reoccurring spots, and then subjected to pixel-by-pixel correlation to the external parameter (i.e., hours of treatment with khat or CPT) using a Spearman rank-order correlation test. The resulting correlation image depicts the correlation to the external parameter with colors varying from red (positive correlation) through black (no correlation) to blue (negative correlation) [22]. Correlation values were collected from defined regions of interest and used to calculate the *t*-value [22], and the probability of significance (*p*-value) was found by calculating the *t*-distribution in a two-tailed Student’s *t*-test with n–1 degrees of freedom.

## 5. Conclusions

This study demonstrated that acidification and accumulation of the p53 FL isoform occurred in khat-sensitive AML cells, whereas the γ/β p53 variants were down-regulated. The p53 isoform modulation was less distinct in the resistant MV4-11 cells, and khat-sensitization by p38 inhibition was not accompanied by stabilization of p53. The results indicate that the p53 isoform pattern could be used as an indirect sensor for chemical cell stressors and experimental therapeutics. We hypothesize that the complex modulations of the p53-network and p53 isoforms provide us with a signature that can be used to explore the involved pathways and molecular mechanisms. 

## Supplementary Materials

The following are available online at https://www.mdpi.com/2072-6694/12/12/3596/s1, Figure S1: Western blot images in Figure 1C, Figure S2: Original figures of Figure 2A, Figure S3: Western blot images in Figure 3A.
