# Supplementary material for: A Comparison of p53 Isoform Profiles and Apoptosis Induced by Camptothecin or a Herbal Khat Extract (Catha Edulis (Vahl) Forssk. ex Endl.) in Leukemic Cell Lines: Exploring Cellular Responses in Therapy Development
Source: Cancers (Basel). 2020 Dec 1;12(12):3596. doi: 10.3390/cancers12123596 (PMC7760143; doi:10.3390/cancers12123596)
Supplement: Supplementary file 1 [file cancers-12-03596-s001.pdf]

Supplementary Materials

A Comparison of p53 Isoform Profiles and Apoptosis Induced by Camptothecin or a Herbal Khat Extract (Catha Edulis (Vahl) Forssk. ex Endl.) in Leukemic Cell Lines: Exploring Cellular Responses in Therapy Development

Therese Bredholt Onyango, Sigrun M. Hjelle, Ingvild Haaland, Olav K. Vintermyr, Anne C. Johannessen and Bjørn T. Gjertsen

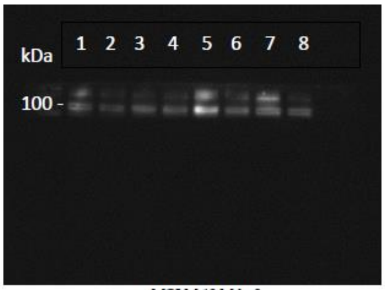

MOLM-13 Mdm2

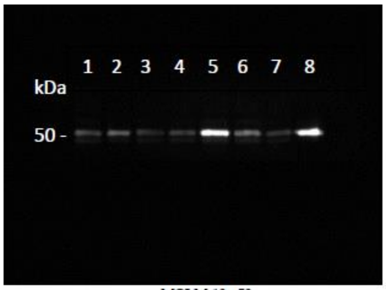

MOLM-13 p53

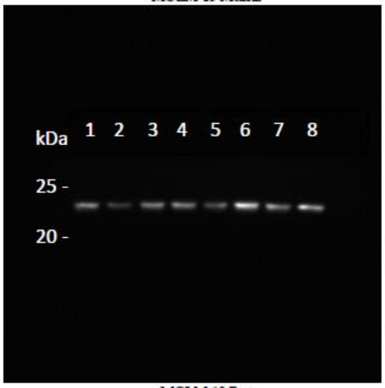

MOLM-13 Bax

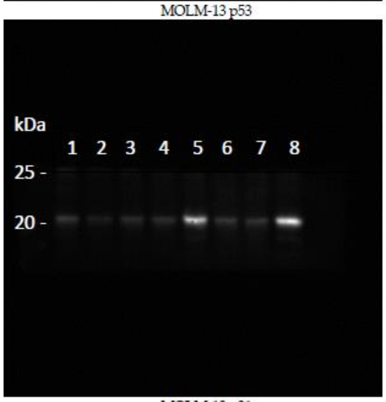

MOLM-13 p21

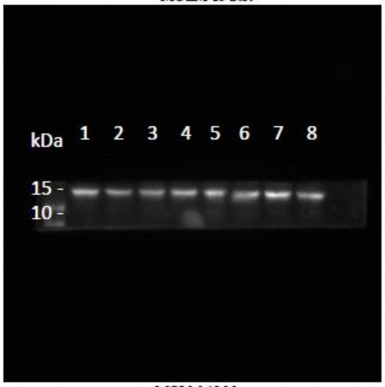

MOLM-13 Noxa

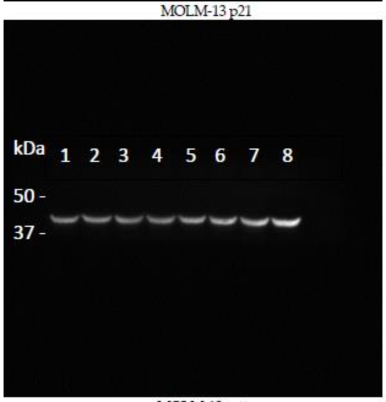

MOLM-13 actin

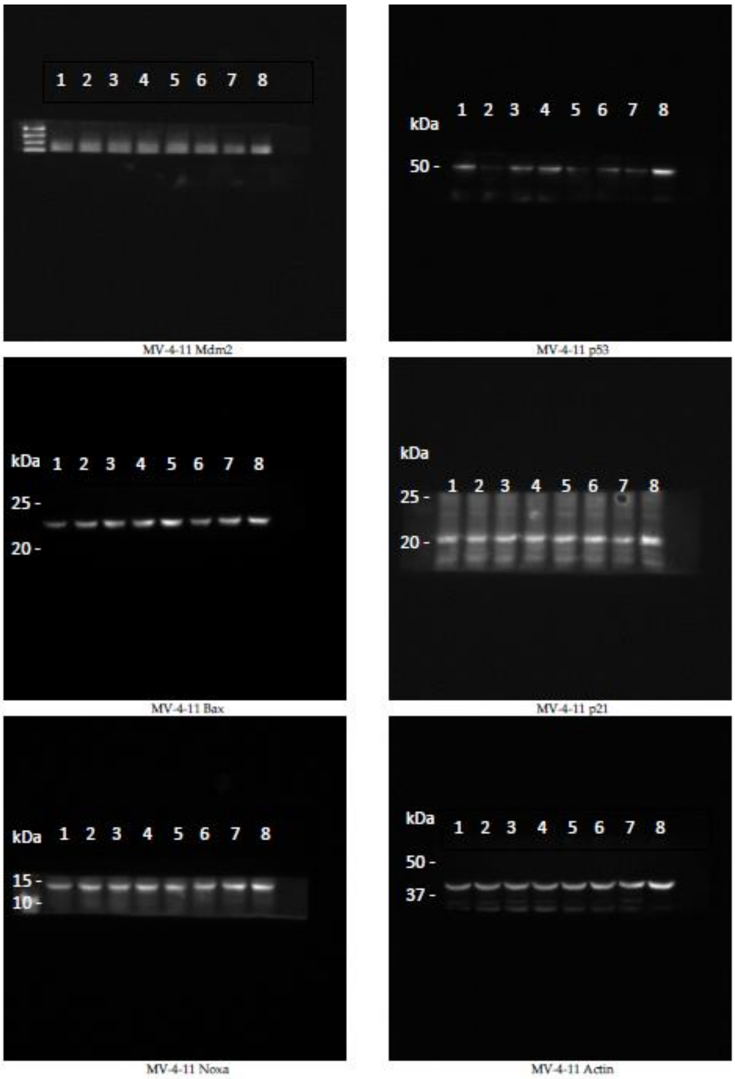

Figure S1. Western blot images in Figure 1C.

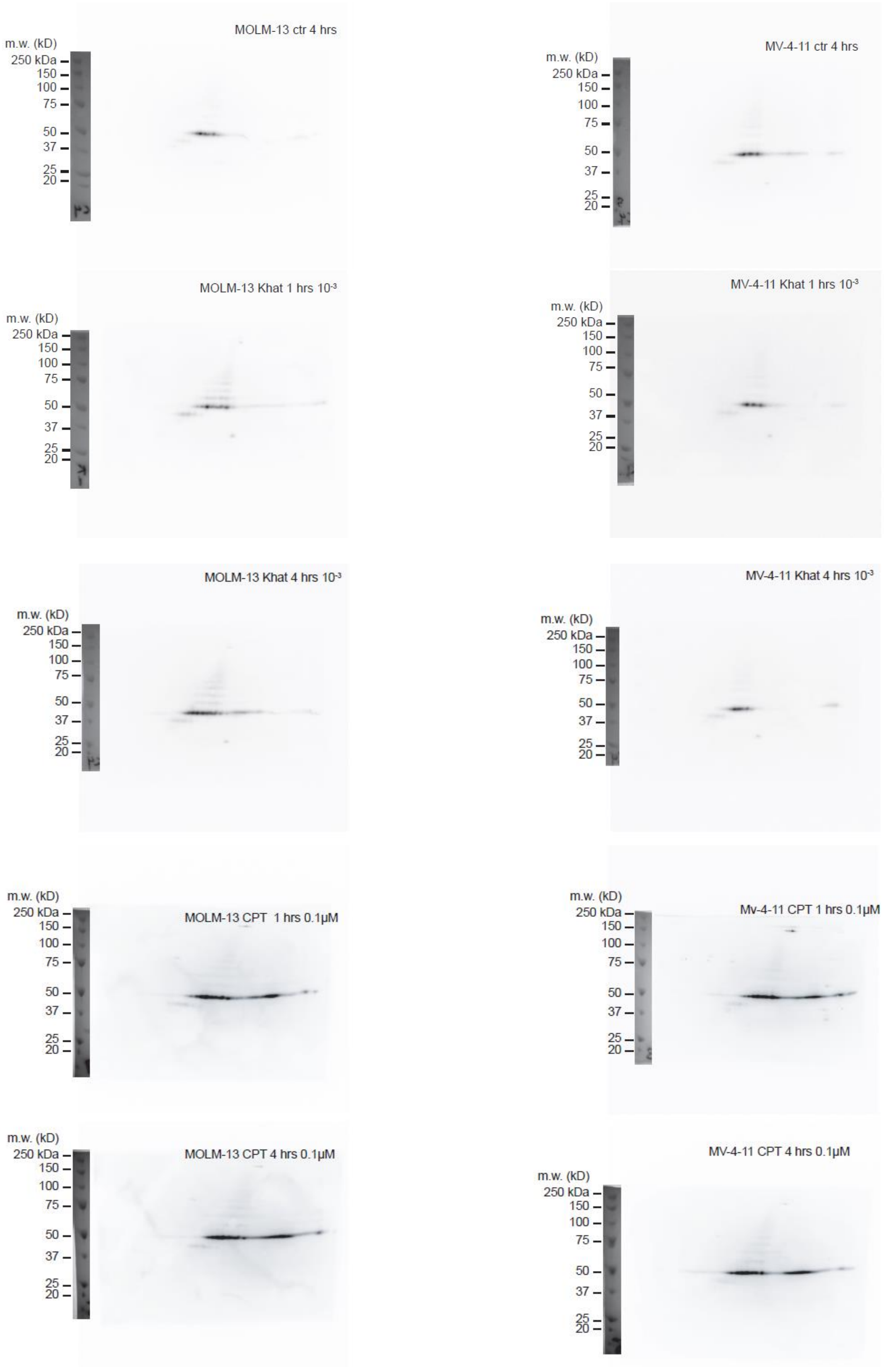

Figure S2. Original figures of Figure 2A.

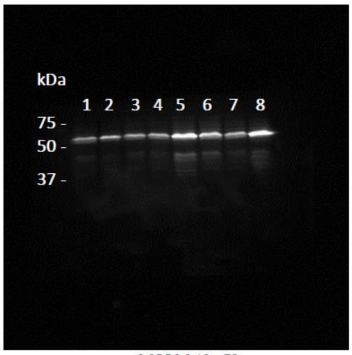

MOLM-13 p53

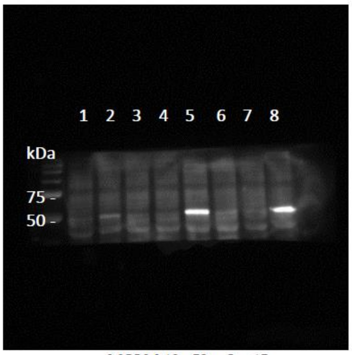

MOLM-13 p53 p-Ser-15

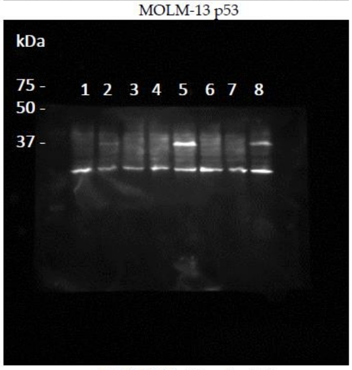

MOLM-13 p53 ac-Lys382

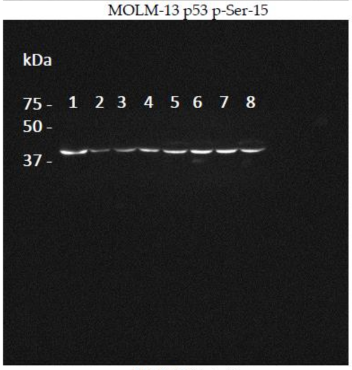

MOLM-13 Actin

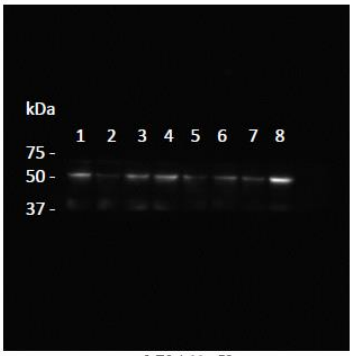

MV-4-11 p53

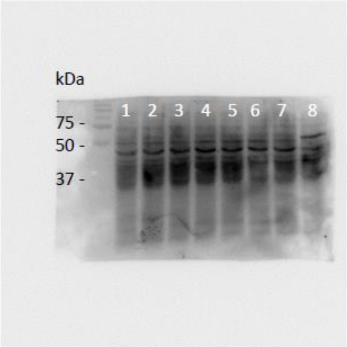

MV-4-11 p53 p-Ser-15

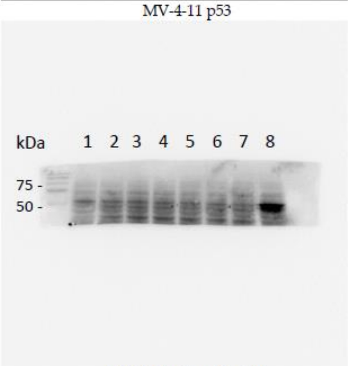

MV-4-11 p53 ac-Lys382

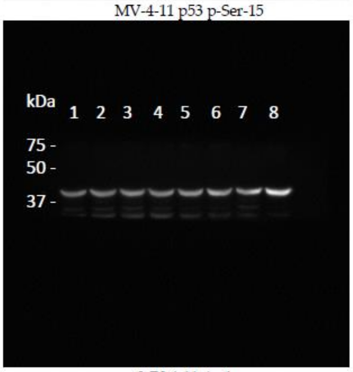

MV-4-11 Actin

Figure S3. Western blot images in Figure 3A.

© 2020 by the authors. Licensee MDPI, Basel, Switzerland. This article is an open access article distributed under the terms and conditions of the Creative Commons Attribution (CC BY) license (http://creativecommons.org/licenses/by/4.0/).
